# Comparing Fragment Binding PosesPrediction Using HSP90 as a Key Study: When Bound Water Makes the Difference

**DOI:** 10.3390/molecules25204651

**Published:** 2020-10-12

**Authors:** Giovanni Bolcato, Maicol Bissaro, Mattia Sturlese, Stefano Moro

**Affiliations:** Molecular Modeling Section, Department of Pharmaceutical and Pharmacological Sciences, University of Padua, 35131 Padua, Italy; giovanni.bolcato.1@phd.unipd.it (G.B.); maicol.bissaro@studenti.unipd.it (M.B.); mattia.sturlese@unipd.it (M.S.)

**Keywords:** fragment-based drug discovery, molecular docking, molecular dynamics, supervised molecular dynamics, water, HSP-90

## Abstract

Fragment-Based Drug Discovery (FBDD) approaches have gained popularitynot only in industry but also in academic research institutes. However, the computational prediction of the binding mode adopted by fragment-like molecules within a protein binding site is still a very challenging task. One of the most crucial aspects of fragment binding is related to the large amounts of bound waters in the targeted binding pocket. The binding affinity of fragmentsmay not be sufficientto displace the bound water molecules. In the present work, we confirmed the importance of the bound water molecules in the correct prediction of the fragment binding mode. Moreover, we investigate whether the use of methods based on explicit solvent molecular dynamics simulations can improve the accuracy of fragment posing. The protein chosen for this study is HSP-90.

## 1. Introduction

Fragment-Based Drug Discovery [1](FBDD) is an ensemble of approaches used in the early stagesofdrug candidates identification which consistsin the screening of small molecules, typically with a molecular weight below 250–300 Da and a logP value below 3 (these empirical criteria are known as the “rule of three” [2]). FBDD approaches have gained popularitynot only in industry, but also in academic research institutes, speeding up the hit-to lead-process and showing an interesting success rate.

Generally, fragment screens lead to the identification of a subset of hit-fragments having an affinity range from μM to mM to the target. However, their identification only represents the beginning of an iterative optimization process to turn a weak fragment into a mature high-affinity lead [3]. One of the challenging aspects of FBDD is the detection of such weak binders commonly achieved by high-sensitivity biophysical techniques, such as isothermal titration calorimetry (ITC), surface plasmon resonance (SPR), thermal shift assay, nuclear magnetic resonance (NMR), and X-ray crystallography (XRC), with only the last two methodologies able to provide structural information.

An alternative to a biophysical method to quickly selecta putative binder from a chemical library is represented by in silicostrategiesin particular when the target structure is available. The virtual screening of fragments is typically a challengingtask; mostly due to the weak performance of scoring functionsusedto discriminate native from non-native poses [3,4].

Most of the scoring functions indeed were mainly developed on high-affinity ligands while fragments are more prone to experience less stable binding states or in certain cases multiple binding modes. For certain targets, the situation can be further complicated by the presence of stable watermolecules within the binding site. In fact, for those cases, theunderstanding of the fragment-target recognition is not only depending on the mere shape or electrostatic and chemical complementarity to itstarget but also the presence of stable solventmolecules. The presence of stable water moleculescan be considered if high-resolution crystallographic structures are available or by computational methodologies investigating the position or the thermodynamic profile of explicit water molecules in protein hot-spots such as 3D-RISM [5], AquaMMapS [6], GIST [7], JAWS [8], SZMAP [9], WaterAlignment [10], WATCLUST [11], WATERDOCK [12], Water FLAP [13], WaterMap [14], and WATsite [15]. Other tools can support the user in selecting those waters that are more stable in high-resolution structures like HINT [16], pyWATER [17], ProBiS H2O [18], WaterScore [19]. It should be noted that the stability of the water network within the protein binding site could be similar to that of weak fragments [20] and, taking this concept to the extreme, a stable water molecule could be considered similar to a very low molecular weight fragment [21]. In this scenario, it is clear that whenever a computational approach is adopted to predict the fragment binding mode an appropriate investigation about the role of the water molecules within the binding site is necessary. From a historical point of view, the first structure-based approach aimed to consider the explicit presence of a water molecule within the binding site was molecular docking [22]. The presence of a stable molecule mediating the ligand interaction may have a great impact on the quality of the pose prediction. Nevertheless, appropriate knowledge about which water molecules to be included is required. The rise of molecular dynamics (MD) strategies which include explicit water offers further alternatives to docking like investigating the stability of a predicted pose along with the monitoring of the water molecules. Novel strategies developed from MD allow investigating the small molecule recognition from the target unbound state with direct observation of the water molecules displaced during the ligand association. One example of thesesimulations is the supervisedmoleculardynamics (SuMD) [23] which allowsus to follow the molecular association in the nanosecond scale without introducing forces or energetical bias.

The present work aims to compare the performance of different methodologies to face the problem of studying the binding mode of fragments in the challengingscenario of a binding site in which stable water molecules are present and play a pivotal role in their stabilization.

Our comparison embraced four different computational approaches: (i) molecular docking without explicit solvent molecules, (ii) molecular docking withhighly conserved water molecules, (iii) molecular docking (without solvent) followed by MD simulation in explicit solvent, and (iv) SuMD starting from the unbound state in explicit solvent.For this study, we have chosen the crystal structures of the loop-in N-terminal domain of Heat.

**Figure 1:**
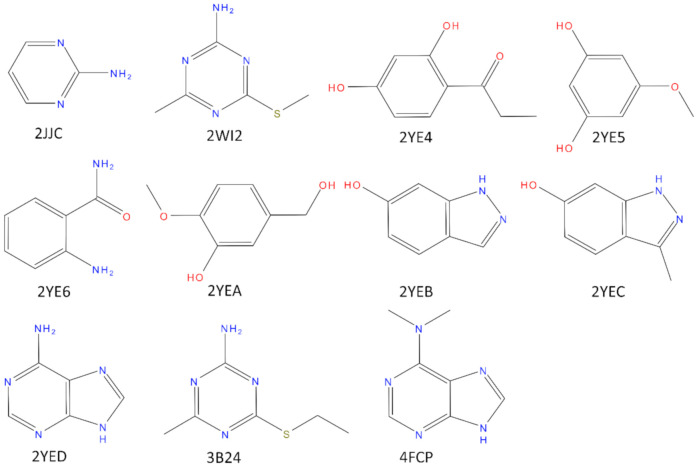
Structure of the crystal ligands bound to N-HSP90 in the structures used for the present work. All the ligands have a molecular weight below 175 Da. Only for complexes 2WIC, 2YE4, and 2YE6 affinity data was reported on literature (2WI2IC_50_ = 350 µM; 2YE4, IC_50_ = 570 µM; 2YE6, IC_50_ = 4000 µM; 3B24, K_d_ = 42 µM).

Shock Protein 90 (N-HSP90) cocrystallized with a low molecular weight ligand (MW < 175 Da). Those crystal structures correspond to the PDB codes: 2JJC, 2WI2, 2YE4, 2YE5, 2YE6, 2YEA, 2YEB, 2YEC, 2YED, 3B24, 4FCP (The structure of the corresponding ligands is reported in Figure 1). We also decided to focus only on the loop-in structures since all the structures of N-HSP90 in apo-form have this specific conformation. HSP90 is a molecular chaperone involved in the maturation of several other proteins and it is a target for the development of chemotherapy agents in many types of cancer. The N-terminal domain of HSP90 binds ATP, essential for the activity of HSP90 [24,25]. The choice of N-HSP90 as a case study is based not only because several high-resolution crystal structures are available for this protein, both in the apo form and in complex with low molecular weight ligand (allowing an accurate study of the structural water molecules), but also due to the well-known role that the solvent plays in the binding between the protein and its ligands [26,27,28]. Interestingly, as pointed out in [29], fragments bind HSP90 through a network of conserved water molecules that mediate the interaction with the protein (in particular with Asn51, Ser52, Asp93, and Gly97). The importance of these structural water molecules in the design of HSP90 inhibitors has been proven [28].

## 2. Results and Discussion

### 2.1. AquaMMapS Simulation Results

Since our comparison includes also MD-based strategies, we first investigated if the conditions used for the MD simulations and the force field chosen wereappropriate to simulate the correct behavior of water.To address this issue, we performed an MD simulation of the HSP90 in the apostate and subjected to AquaMMaps [6] toassess if the regions with predicted stationary water molecules were in agreement with the position of those having low B-factor observed in X-ray structures.

AquaMMapsisa software that, through a posteriorianalysis of water molecule trajectories during explicit solvent molecular dynamics simulations can calculate for each space region an occupancy value that expresses the ratio between the time during which a water molecule is located in that region during the dynamics and the total time of the simulation.

**Figure 2:**
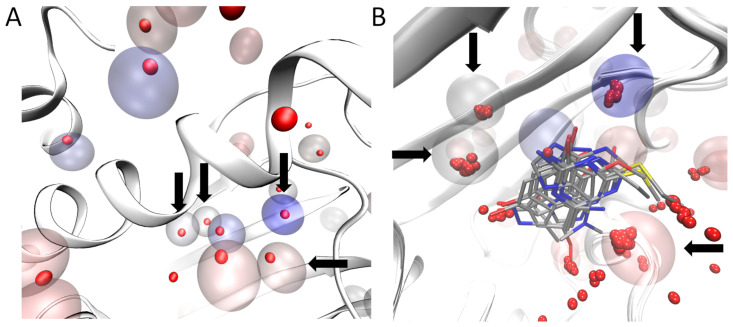
Identification of water molecules within the binding site of N-HSP90 (PDB ID: 5J2V). Panel (**A**): The red opaque spheres correspond to the oxygen atom of the crystallographic water with a low B-factor (below 25) while the transparent spheres are the cell predicted by AquaMMapS with a high %O_RMSF_ value (above 25), the color of the AquaMMapS cell refers to the %O_RMSF_ value of that cell (the %O_RMSF_ value increases from red to blue). As it can be observed, there is a good agreement between the high occupancy AquaMMapS cells and the low B-factor crystallographic water molecules. Panel (**B**): Superposition of the 11 crystals structures used in this work. The Four highly conserved water molecules identified by pyWATER are marked by the black arrows. Each highly conserved water molecule corresponds to a high %O_RMSF_ valueAquaMMapS cell (the cells displayed have all an %O_RMSF_ value greater than 25).

The AquaMMapS analysis was performed for five replicates of 100 ns for a total simulation time of 500 ns.These five replicates were merged and submitted to the AquaMMapS analysis. As reported in Figure 2, a good agreement as observed between the AquaMMapS cells with a %O_RMSF_ value greater than 25 (see Materials and Methods for a detailed explanation of AquaMMapS and its outputs) and the crystallographic waters with a B-factor below 25, especially within the binding site. The crystal structure employed for these simulationsis5J2V, this is one of the several structures of N-HSP90 in apo form.

### 2.2. Docking, MD Post-Docking, and SuMD Simulation Results

Four different approaches were set up to assess their ability in reproducing the crystallographic structure by evaluating the RMSD: (i) molecular docking without explicit solvent molecules, (ii) molecular docking with highly conserved water molecules, (iii) molecular docking (without solvent) followed by MD in explicit solvent (post-docking MD), and (iv) SuMD starting from the unbound state in fully explicit solvent.

Thedockingsimulationshave been performed using GOLD 5.4.1 with the Chemscore scoring function. We identified this protocol by comparing the performance of 17 different docking protocols over all the available ligand-HSP90 complexed reported in the protein data bank (see details supporting informationand Figure S1). Starting from this benchmark, we focusedour attention on the results for the 11test cases selected in this work. Among allothers, Gold-based protocolsoutperformed the other ones. Finally, we restricted the comparison on the two scoring functionson which the implementation of water molecules has been reported: goldscore and chemscore [30]. Besides the identical dockbench cumulative score of the two (both scored 5, see Table S1 on SI), chemscore showed better results in terms of minimum RMSD averaged on the 11 structures (goldscore:2.64Å; chemscore: 1.64 Å) and hence selected for docking calculation. In the firstapproach (i), the crystallographic ligands have been docked within the binding site of N-HSP90 without any water molecule. On the contrary, in the second method, the same docking protocol was implemented to include four water molecules placed in the binding site. These four water molecules have beenchosen as they appear to be highly conserved in all the structures employed in this work. To address the challenging issue in identifying which water molecules to include in the calculation among those experimentally reported on the test set, we decided to adoptpyWATER tool [17]. This method identifiesstable water molecules by a consensus strategythrough a cluster-based approach. For each docking protocol, (i) and (ii), three poses have been generated for each ligand.

**Figure 3:**
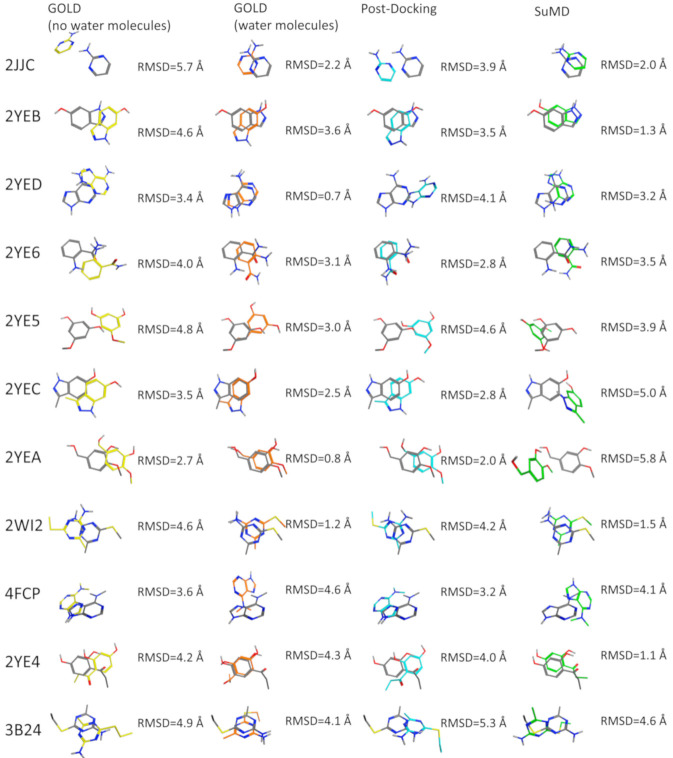
First pose comparison between the different methods (yellow: GOLD without water molecules. Orange: GOLD with water molecules. Blue: Post-Docking. Green: SuMD) for each fragment. In gray is reported the crystallographic pose.

**Figure 4:**
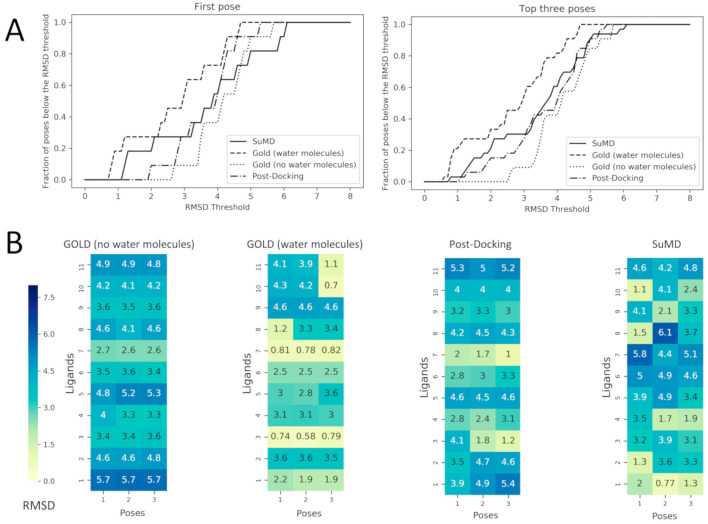
Performance comparison between Docking with and without the consideration of conserved water molecules within the binding site and the two MD based approach: post-Docking and SuMD. These comparisons are made calculating the RMSD values of the predicted poses respect to the crystallographic ones. In Panel (**A**) the fraction of poses below an RMSD threshold are displayed as a function of the threshold itself, this analysis is reported both for the first pose (the one with the best score value for Docking and with the best MMGBSA value for SuMD) and for the top three poses (always the three with the best score or MMGBSA values). In Panel (**B**) the RMSD values of each pose are reported for each ligand as heatmaps. The poses are ordered from left to the right according to their score values for Docking and to their MMGBSA values for SuMD and for post-Docking (so pose 1 has a better score/MMGBSA value in comparison with pose 2 and so on), the heatmap is colored according to the RMSD value which is reported in each grid box. Tobetter compare docking strategies, (i) and (ii), which resulted in three poses, also for MD-based protocol (the post-docking MD and SuMD), three poses for each fragment have been selected by adopting a clustering strategy to select significant representative fragment conformation among the trajectory frames and ranked by the MMGBSA method.

The post-docking MD strategy started with the best pose obtained from the docking withoutsolvent. The pose was hence equilibrated in a fully explicit solvent simulation box and thesystem was finally refined by classical MD for 25 ns. This approach aimed to observe if the roleof the solvent missing in the docking calculation could be eventually restored by an a posteriori strategy. The advantage of this strategy is that a priori information about the stable water molecules is not required. On the contrary, the drawback of an a posteriori strategy could be eventually the steric hindrance of the ligand placed in the binding site that could obstruct the correct placing of the waters. In light of this hypothesis, the fourth protocol was based on a more demanding strategy simulating the recognition event of a fragment from the unbound state by using SuMD. In this protocol, the fragment was placed 30 Å away from its HSP90 binding site. In this way, the binding site is fully solvated by explicit water and the ligand needs to displace them during the recognition. To better understand the SuMD methodology, a recognition trajectory for the complex HSP90–2-pyrimidinamine (PDB ID: 2JJC) is rpresentedin Video-S1. The fragment nicely displaced the solvent in the HSP90 cavity but the regions characterized by stable water molecules are not explored where the water molecules are retained and they mediate the interaction with the fragment and HSP90 in a very similar way to the experimentally solved complex.The comparison between the first pose for the fourprotocols is reported in Figure 3. To go into more detailed comparison, in Figure 4 (panel B), the RMSD value of each pose for each ligand is reported, the poses are ordered according to their docking score and to their MMGBSA value (for docking-based and MD-based, respectively). The RMSD values were further used to measure the ability four protocols in geometrically reproducing the experimental complex; in panel A we reported the relationship between the fraction of poses reproduced (below a certain RMSD) to the RMSD. We performed this analysis for both for the first pose and the top-three poses. Ideally, the sooner the profile reaches the top of the fraction of poses reproduced respect to RMSD better the protocol is performing. It should be noted that for the considered complexes we observed that the poses with RMSD lower than 2 Å showed also a correct pattern of interaction with the target, in particular presenting the key interaction with Asp93.

The most clear results are about the performance of the docking protocol without water molecules which results are poor; this can be expected since this is a challenging scenario for a docking protocol not only for the low molecular weight of the ligands but also for the use of the apo form of the protein. The best scoring poses for this protocol report high RMSD values spanning from 2.7 to 5.7 Å; in this range of RMSD the specific fragment-protein molecular interactions observed in the experimentally solved complex are lost. Instead, a dramatic increase in the performance of docking posing is observed simply retaining the four aforementionedwater molecules in the calculation. When the four water molecules are taken into account the performance notably increases, and most importantly the binding mode of the fragments 2YED, 2YEA, 2WI2 is correctly predicted (the RMSD of best pose for these complexes drops below 1.2 Å).

The performance of molecular docking without explicit water molecules is enhancedwhen coupled with a post-docking refinement of thebest scoring pose. The refinement of the docking pose lead to a lower RMSD value for every fragment except for 3B24. It should be noted that despite the improvement in terms of RMSD only in one case—2YEA—in which the hydrogen bond with the Asp93 is restored, was the correct binding mode recovered.Despite the improvement due to the refinement procedure the results do not reach the quality of the docking protocol with the explicit water molecules.

Among the MD-based protocols, SuMD (iv) outperformed post-docking MD both in terms of RMSD and in the binding mode sampling. In Video-S2 (Supplementary Material) the superposed trajectories of SUMD runs are reported to highlight the association process of the fragments to the fully solvated HSP90 binding site. The performance of SuMD in terms of RMSD seems to be superioralsoto the molecular docking without water molecules (i) but slightly below to GOLD retaining the fourcrystallographic water molecules (ii). The poses of 2JJC, 2YEB, 2WI2, 2YE4 have been predicted by SuMD with an RMSD value below 2Å with respect to the crystallographic pose and most importantly for those fragments, the binding mode is correctly predicted by SuMD. It is interesting to note that despite the lower performance respect to the docking with the explicit water molecules (ii) from a geometric point of view (i.e., RMSD), SuMD (iv) is slightly better in reproducing the correct binding mode: four complexes for SuMD whileonly three complexes GOLD with explicit solvent (ii).

A further notable observation is that all the four protocols failed to reproduce the posesfor 2YE6, 2YEC, 4FCP, and 3B24. This observation indicates that fragment pose prediction still represents a challenging task, even for advanced structure-based approaches. Also, we observed that the docking differently performed depending on the test case (i.e., the complexes correctly reproduced are different for each protocol), suggesting that it is difficult to have a clear picture in the identification of the most appropriate protocol a priori.

The case of 3B24 and 2WI2 is particularly interesting. The two fragments differ only ina methylene group: 3B24 presents an ethyl group attached to the sulfur atom while 2WI2 has a methyl group. The crystallographic binding mode of the two fragments is very well conserved but the pose prediction performance of the different protocols is quite different. In the case of 2WI2, both molecular docking with water molecules (ii) and SuMD (iv) reproduced the crystallographic pose with an RMSD tolerance of 1.2 Å and 1.5 Å, respectively. On the contrary, in the case of3B24, all protocols fail in the pose prediction with RMSD values over 4 Å. Surprisingly, the affinity reported for 3B34 is particularly high (K_D_ = 42 µM) for such a small fragment and the resolution of the complex is higher than 2WI2 (1.70 Å and 2.09 Å, respectively).

It is clear that the molecular docking in presence of defined and explicit water moleculesoutperformed in terms of RMSD the other approaches followed by a more time-demanding SuMD method that on the contrary did not require a priori information about the stable solvent molecules.We observed that for the correctly predicted case SuMD not only nicely reproduced the bound-state geometries, but the stable hydration sites were also retained (Video-S1). Thisaspect represents the most notable advantage of SuMD and, in perspective, it could be particularly relevant for all those cases in which a few information is available about the role of the solvent in mediating the ligand-protein interaction. MD-based refinement slightly improves the performance of the docking without water molecules but is not able to balance the performancesneitherof docking with water molecules nor SuMD.

A further aspect that should be considered in the comparison of those methodologies is the different calculation time required. While for molecular docking a single run can be performed on the order of minutes, MD-based approaches are more demanding and to complete a SuMD simulation usually requires around a dozen hours to complete on a modern GPU card. On the same hardware, a post-docking MD refinement can be easily achieved within a couple of hours. Finally, it should be also considered that the four different protocols present a different level of complexity. Undeniably, molecular docking protocol is easier to set up in comparison to molecular dynamics, and as a consequence, it is suitable for a larger number of users.

## 3. Materials and Methods

### 3.1. System Preparation and MD Setup

System preparation has been performed using the Molecular Operating Environment (MOE) suite [31] for what concerns the protein preparation (removing the crystallographic water molecules, ions, and other solvents, selecting of the highest occupancy alternate for each residue, assigning the correct protonation state at pH 7.4 to all atoms). The system preparation for the Molecular Dynamics Simulations has been carried out using AmberTools14 [32,33] for what concerns the simulations performed with the ff14SB force field. The protein was explicitly solvated in a water box with the borders placed at a distance of 15 Å from any protein atom, the water model used was TIP3P [34]. The system charge was neutralizedto a concentration of 0.154 M using Na^+^/Cl^−^.

Molecular dynamics simulations have been performed using ACEMD [35]. The system energy was minimized in 500 steps using the conjugate-gradient method, then, during the equilibration stage, two simulations have been done. The first consistedof 0.1 ns of NVT simulation with harmonic positional constraints of 1 kcal mol^−1^Å^−2^ on each protein atom. The second consistedof 0.5 ns of NPT simulation with harmonic positional constraints of 1 kcal mol^−1^Å^−2^ only on the α-carbons of the protein. The simulations consist of 100 ns NVT simulations (temperature 310 K, timestep 2 fs), the last 50 ns of these simulations were submitted to the AquaMMapS analysis.

### 3.2. AquaMMapS

AquaMMapSis a software aimed to identify hydration sites at the protein-solvent interface in which water molecules show a high-occupancy rate during an MD simulation. Briefly, the tool performs a grid-based analysis of the frequency of occupation of the water molecules. The size is chosen to accommodate one water molecule per cell at most. For each cell of the grid two data are computed: an occupancy value that expresses the ratio between the number of frames during which that cell has been occupied by a water molecule and the total number of frames (%Oall), and an occupancy value that expresses instead the ratio between the number of frames during which that cell has been occupied by a stationary water molecule (i.e., water molecules with an RMSF below1.4Å) and the total number of frames (%O_RMSF_), so if a cell has an %O_RMSF_ of 25%, this means that during the simulation this cell has been occupied by a stationary water molecule for 25% of the frames.

### 3.3. Molecular Docking

A benchmark of 17 different docking protocols over 200 HSP90-ligand x-ray complexes was performed using DockBench [36] to select the most suitable protocol (details are provided in the Suplementary Material). The results were in agreement with previously reported docking studies on HSP90 [37].

GOLD 5.4.1 was used asdockingengineandcoupled to the scoring function Chemscore. GOLD is a flexible docking protocol that relies on a genetic algorithm for the pose generation while Chemscore is an empirical Scoring Function [38].

The center of the binding site has been defined by superposing all the structure on 2JJC andusing the center of mass of its crystallographic ligand.Two docking runs have been performed, one with the inclusion of water molecules within the binding site and one without those water molecules. For each run three poses for each fragment have been generated with an RMSD clustering value of 2 Å. In the docking run with the inclusion of the water molecules, these have always been present (*on* option), the position of the oxygen atom is fixed while the position of the hydrogen is optimized by GOLD (*spin* option).

The clustering analysis on the holo loop-in crystal structures of HSP90 has been performed using the tool PyWATER [17]. PyWATER works aligning a series of protein crystal structures of interest and performing a clustering analysis on the crystallographic water molecules to identify the most conserved water molecules among the different crystals.

Four highly conserved water molecules have been detected and retained in the protein structure for the Docking calculation. The four water molecules correspond toresidues 2078, 2082, 2164, and 2166 in the PDB entry 2JJC.The orientation of the water molecules is optimized by GOLD for each case.

### 3.4. SuMD Simulations, Post-Docking Simulations, and Pose Selection

SuMD [39,40] is a method based on MD aimed to investigate molecular recognition events without energetic biases. Briefly, the algorithm relies on thesupervision of the ligand-protein center of mass distance during consecutive small classical MD simulation. The supervision algorithm acts at the end of each small simulation, named SuMD step: if this distance is likely to be shortened during the SuMD step, thesimulation is prolonged by a further SuMD step, otherwise, it is stopped, and thesimulation is restarted from the previous set of coordinates. In this work, fragments were placed 30 Å away from the protein. Each SuMD step was set to 300 psThe default settings were maintained except the maximum number of consecutive rejected SuMD step that was set to 30. At the end of the SuMD process, the simulation has been extended for 25 ns of classical MD.

The threeconformationsreported for each fragment have been selected as follows. For each case study, ten SuMD simulations have been performed and only the simulations which led to a binding event have been retained (so only the simulation in which the 30 classical steps of MD below 5 Å has been performed). These trajectories have been aligned on the same reference and merged. The position of the ligand in the merged trajectory has been clustered using Scikit-learn [41]. First, all the sets of coordinates of the ligand (each set is composed of the coordinates of the ligand in a frame of the trajectory) identified as noise by the OPTICS algorithm have been discarded, then all the remaining set of coordinates have been clustered using K-means. The number of clusters has been set to three, in analogy to the threeposes obtained in the docking calculation, and to facilitatethe comparison with this. For each cluster, the set of coordinates identified as a centroid has been selected as representative of that clusterand then the threecentroids obtained for each fragment have been ranked according to their MMGBSA value.

For what concerns the post-docking refinement, three simulations of 25ns for each fragment have been performed on the best pose resulting from the docking calculation with GOLD (without water molecules within the binding site). The simulations have been performed with the same conditions used for the SuMD simulations. The three trajectories for each fragment have been aligned and merged, then three poses have been extracted as described above for SuMD.

## 4. Conclusions

The results of the present work emphasize once again the importance of taking into account structural water molecules in the prediction of fragment binding modes. We have focused our investigation on small molecular weight ligands for which molecular docking protocols usually exhibit poorer performance than with classical high-affinity ligands.As expected, the docking simulation carried out without any structural water resulted in poor results; this observation was in agreement with our previous observation that for HSP90 the absence of stable water molecules deteriorates the docking performances even for ligand with stronger affinity [37]. On the contrary, when the conserved water molecules within the binding site are retained or more sophisticated methods like SUMD have used the performances increase dramatically.

For the protein under investigation, N-HSP90, several crystal structures are available, so the identification and the placement of conserved water molecules is an easy task. In this scenario, molecular docking with specific water mediating the interaction remains the best choice both in terms of computational effort and in geometrical terms, but in the worst-case scenario where no information about water molecules isavailable, likein the case of low-resolution XRC structures or for NMR-based ones, the use of explicit solvent MD simulation can be useful to fill this gap from several points of view. First, one could place stable water molecules using MD-based tools, several tools are already designed with this aim.Afurther possibility is investigating the ligand recognition process starting from a distant position of the ligand; the role of stable water molecules could be restored since the ligand will need to displacemost of the solvent present in the binding sitebut maintain those water molecules that guarantee a more stable interaction or that are less prone to be displaced. Finally, a slight improvement of pose prediction could be obtained by performing the post-docking refinement of the docking pose, the results are better than those obtained with Docking when no water molecules are considered.

## Supplementary Materials

Video S1: SuMD simulationof the binding event of pyrimidinamine. The crystal structure is 2JJC. Video S2: Superposed trajectories of different SuMD simulations.
