# Supplementary material for: Comparing Fragment Binding PosesPrediction Using HSP90 as a Key Study: When Bound Water Makes the Difference
Source: Molecules. 2020 Oct 12;25(20):4651. doi: 10.3390/molecules25204651 (PMC7587341; doi:10.3390/molecules25204651)
Supplement: Supplementary file 1 [file molecules-25-04651-s001.zip › SMoro_Molecules_SI.docx]

Comparing fragment binding poses prediction using HSP90 as a key study: when bound water makes the difference.

Giovanni Bolcato^1^, Maicol Bissaro^1^, Mattia Sturlese^1^ and Stefano Moro^1,^*

^1^ Molecular Modeling Section, Department of Pharmaceutical and Pharmacological Sciences, University of Padova, 35131 Padova, Italy

***** Correspondence: stefano.moro@unipd.it

**Supporting Materials**

Video-S1 caption. SuMD simulation of the recognition event for the complex PDB ID: 2JJC. The crystallographic pose of the fragment is reported in grey. The transparent spheres represent the high occupancy cells by water molecules previously identified by AquaMMapS. The explicit water molecules populating these cells during the simulation are also displayed.

Video-S2 caption.Superposition of all the recognition trajectories generated by SuMD in the first Virtual Screening. All the recognition trajectories of the (10 replicas each) were superposed based on the Ca protein atoms and aligned to the first trajectory frame. Only one protein trajectory is shown for clarity (white ribbon).
